# Change in Oxidative Stress Biomarkers During 30 Days in Saturation Dive: A Pilot Study

**DOI:** 10.3390/ijerph17197118

**Published:** 2020-09-28

**Authors:** Simona Mrakic-Sposta, Alessandra Vezzoli, Federica D’Alessandro, Matteo Paganini, Cinzia Dellanoce, Danilo Cialoni, Gerardo Bosco

**Affiliations:** 1 Institute of Clinical Physiology, National Research Council (CNR), 20162 Milan, Italy; simona.mrakicsposta@cnr.it (S.M.-S.); alessandra.vezzoli@cnr.it (A.V.); cinzia.dellanoce@ifc.cnr.it (C.D.); 2 Department of Biomedical Sciences, Environmental and Respiratory Physiology, University of Padova, 35122 Padova, Italy; fe.daledoc@libero.it (F.D.); dcialoni@daneurope.org (D.C.); gerardo.bosco@unipd.it (G.B.); 3 DAN Europe Research Division, 64026 Roseto degli Abruzzi, Italy

**Keywords:** saturation diving, hyperbaria, reactive oxygen species, nitric oxide, electron paramagnetic resonance

## Abstract

Saturation diving allows divers to reduce the risk of decompression sickness while working at depth for prolonged periods but may increase reactive oxygen species (ROS) production. Such modifications can affect endothelial function by exacerbating oxidative stress. This study investigated the effects of saturation diving on oxidative stress damage. Redox status was evaluated through: ROS production; total antioxidant capacity (TAC); nitric oxide metabolites (NOx); nitrotyrosine (3-NT); and lipid peroxidation (8-iso-PGF2α) assessment. Creatinine and neopterin were analyzed as markers of renal function and damage. Measurements were performed on saliva and urine samples obtained at four time points: pre; deep; post; and 24 h post. Four divers were included in the study. After the saturation dive (post), significant (*p* < 0.05) increases in ROS (0.12 ± 0.03 vs. 0.36 ± 0.06 µmol.min^−1^), TAC (1.88 ± 0.03 vs. 2.01 ± 0.08 mM), NOx (207.0 ± 103.3 vs. 441.8 ± 97.3 µM), 3-NT (43.32 ± 18.03 vs. 18.64 ± 7.45 nM·L^−1^), and 8-iso-PGF2α (249.7 ± 45.1 vs. 371.9 ± 54.9 pg·mg^−1^ creatinine) were detected. Markers of renal damage were increased as well after the end of the saturation dive (creatinine 0.54 ± 0.22 vs. 2.72 ± 1.12 g-L^−1^; neopterin 73.3 ± 27.9 vs. 174.3 ± 20.53 μmol·mol^−1^ creatinine). These results could ameliorate commercial or military diving protocols or improve the understanding of symptoms caused by oxygen level elevation.

## 1. Introduction

Saturation diving, also known as “Commercial Offshore” or “Diving Underwater Habitat”, is a prolonged diving technique that brings gas concentrations in tissues into equilibrium with environmental gases [1]. These divers work at great depths for a long time and live inside underwater habitats to carry out essential human functions. During saturation diving, the human body is subjected to severe/extreme environmental conditions, exposing divers to higher risks and accidents [2,3]. However, the risk of decompression sickness is reduced by controlling the partial pressures of inhaled gases [2]. The responses to stressful conditions have been documented using various physiological, biochemical, and psychological measures [4,5,6]. Specifically, divers are exposed to increased partial pressure of oxygen (PO_2_), potentially toxic gases, bacteria, and bubble formation during decompression. These subjects also spend a long time in isolation, alternating strenuous shift work with prolonged relative inactivity [7].

Under normal physiological conditions, approximately 1.2% of inspired O_2_ is converted to reactive oxygen species (ROS), and hyperoxia increases this amount [7]. As suggested by previous works on hyperbaric hyperoxic exposure in mammalians [8,9,10] challenging variations in PO_2_ are known to induce inflammation [9,11,12] and increased ROS production. These byproducts exacerbate oxidative stress and damage cell structures, specifically proteins, lipids, and nucleic acids [13,14].

Little is known about the balance between pro-oxidant effects and antioxidant responses related to saturation diving. Antioxidant defenses are known to increase when the production of ROS is enhanced [7,15,16]. Additionally, the administration of antioxidant and vitamin supplements is an intuitive measure to boost antioxidant defenses against ROS [17]. Recent literature has demonstrated that differential gene expression indicated reduced blood O_2_ transport and increased endogenous antioxidant activity after saturation diving.

Another O_2_ free radical produced as part of normal physiology is nitric oxide (NO). Hyperoxia increases the activity of all three NO Synthase isoforms (iNOS: inducible; eNOS: endothelial; and nNOS: neuronal) via several mechanisms [18,19]. NO seems to play a vital role in decompression stress, but the specific interactions during saturation diving are still unknown.

This study aimed to investigate the effects of saturation diving on oxidative stress for the first time. In particular, we planned to determine ROS production and total antioxidant capacity (TAC) via electron paramagnetic resonance (EPR), the only technique that enables the direct detection of free radicals [20,21,22], using a specific spin probe [22,23]. Moreover, NO metabolites and nitrotyrosine (markers of cell damage and inflammation), interleukin 6 (IL-6) and 8-isoprostane (marker of lipid peroxidation), and creatinine and neopterin (markers of renal function and damage) were evaluated.

## 2. Material and Methods

### 2.1. Subjects, Worksite, and Experimental Design

This study involved four professional (certified for commercial saturation diving) divers working in the Southern Adriatic Sea, whose anthropometric and physiological parameters are reported in Table 1.

The study was conducted following the Helsinki Declaration and was approved by the Ethical Committee of Università degli Studi di Milano, Italy (Aut. n° 37/17). All the volunteers signed an informed consent. Monitoring sessions were conducted onboard the main ship between 20 December 2019 and 21 January 2020.

**Figure 1:**
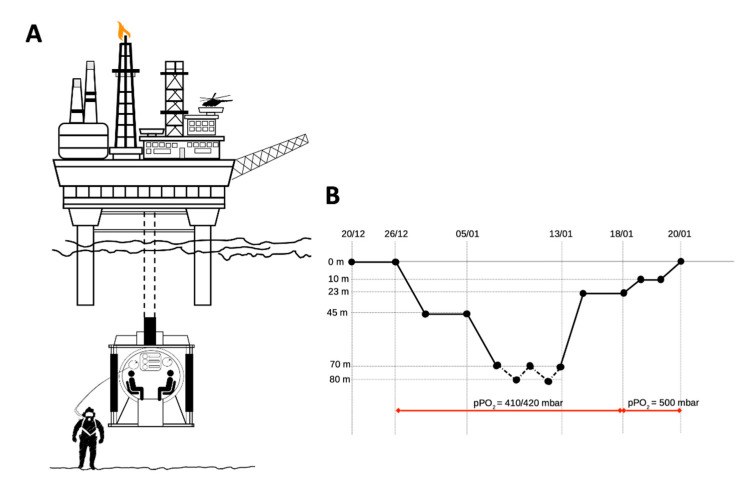
Experimental study design. In (**A**) the accommodation chamber; (**B**) diving profile: divers were onboard from 20 to 26 December, then were compressed to 45 m and worked there until 5 January. Then they were compressed to 70 m and worked between 70 and 80 m for about 8 days. Finally, divers were progressively decompressed to surface (with a brief stop at 23 m for potential supplemental work at that depth, not performed).

In Figure 1, the diving profile is depicted. At maximum depth, PO_2_ was kept between 410 and 420 mbar, increasing to 500 mbar at the end of the decompression. The hyperbaric environment had a controlled humidity of 50–70% and a temperature of 29–31 °C. Additionally, carbon dioxide was monitored continuously to ensure a maximum level of 5000 ppm. Divers alternated 8–10 h of underwater work with 12 h of rest in the chamber hosted onboard. While working at depth, the PO_2_ of the lockout gas supplied to the divers was set between 0.6 and 0.9 atm.

Antioxidant supplements (see Table 2) were administered to the divers to increase the body’s antioxidant defenses.

Samples of saliva and urine were obtained at four time points before, during, and after a saturation dive: before (pre) on 20 December; at maximum depth on 10 January (deep); immediately after the end on 20 January (post); and 24 h after the end on 21 January (24 h post).

### 2.2. Saliva and Urine Samples

Saliva samples were collected to determine levels of ROS and TAC. Nitrite/nitrate (NOx), 3-nitrotyrosine (3-NT), lipid peroxidation (8-isoprostane), IL-6, neopterin, and creatinine concentrations were measured on urine samples. 

Approximately 1 mL of saliva was obtained by Salivette devices (Sarstedt, Nümbrecht, Germany). The subjects were instructed on the correct use and to refrain from drinking, eating, smoking, brushing their teeth, and using mouthwash in the 30 min before salivary collection. Samples were spun down, aliquoted, and stored.

Urine samples were collected by voluntary voiding in a sterile container and were stored in multiple aliquots at −20 °C until assayed and thawed only once before analysis. 

#### 2.2.1. ROS and Antioxidant Capacity by Electron Paramagnetic Resonance

Electron Paramagnetic Resonance spectroscopy X-band (9.3 GHz) (E-Scan Bruker, Billerica, MA, USA) was used to assess ROS production and TAC. Samples were analyzed in triplicate. A 37 °C unit by Temperature and Gas Controller ‘‘Bio III’’ (Noxigen Science Transfer & Diagnostics GmbH, Elzach, Germany), interfaced with the E-Scan, was preserved. ROS production and TAC assessment methods were previously described [14,23,24,25,26].

#### 2.2.2. Nitrite and Nitrate Levels (NO_x_)

NOx concentrations were assessed in urine via a colorimetric method based on the Griess reaction [27], using a commercial kit (Cayman, BertinPharma, Montigny le Bretonneux, France). Samples were read in duplicate at 545 nm by a spectrophotometer microplate reader (Infinite M200, Tecan Group Ltd., Männedorf, Switzerland). This method has been previously described [14,27].

#### 2.2.3. Nitrotyrosine (3-NT)

3-NT and NO levels have a direct relationship. The assay kit adopted (cat no EU2560; FineTest, Wuhan, China) used Competitive-ELISA as the assessment method. The analysis was carried out in accordance with the manufacturer’s instructions. The concentration of 3-NT in urine was measured spectrophotometrically at a wavelength of 450 nm by comparing the samples’ OD ( optical density) to a standard curve.

#### 2.2.4. 8-Isoprostane

Lipid peroxidation was assessed in urine by competitive immunoassay of 8-isoprostane concentration (8-iso-PGF2 α) (Cayman Chemical, Ann Arbor, MI, USA). Samples were read in duplicate at a wavelength of 512 nm. This method has been previously described [12,14].

#### 2.2.5. Interleukin-IL-6

IL-6 levels were determined using the ELISA assay kit (ThermoFisher Scientific, Waltham, MA, USA), based on the double-antibody “sandwich” technique in accordance with the manufacturer’s instruction.

All the above samples and standards were read by a microplate reader spectrophotometer (Infinite M200, Tecan Group Ltd., Männedorf, Switzerland). The determinations were assessed in duplicate, and the inter-assay coefficient of variation was in the range indicated by the manufacturer.

#### 2.2.6. Creatinine and Neopterin Concentration

Urinary creatinine and neopterin concentrations were measured by isocratic high-pressure liquid chromatography (HPLC). Methods have been previously described [12,14].

### 2.3. Statistical Analysis

Data are presented as mean ± standard deviation (SD). Statistical analysis was performed using the GraphPad Prism package (GraphPad Prism 8. 4. 3, GraphPad So ware Inc., San Diego, CA, USA). After the Shapiro–Wilk normality test, statistical analyses were performed using non-parametric tests. Data were analyzed with the Friedman non-parametric test for multiple comparisons and Dunn’s post-hoc to determine differences among conditions. *p* < 0.05 was considered statistically significant. Change ∆% estimation (((post value-pre value)/pre value) × 100) is also reported in the text.

## 3. Results

No significant differences were observed in anthropometric and physiological parameters between pre and post 30 days of saturation diving. Only a small percentage reduction in weight (−4%), measures of: the waist (−2%) and hips ( anatomic part of the body) (−1.5%), BMI (−4%), and an increase in diastolic blood pressure (DBP) (+13%) can be observed (see Table 1).

**Figure 2:**
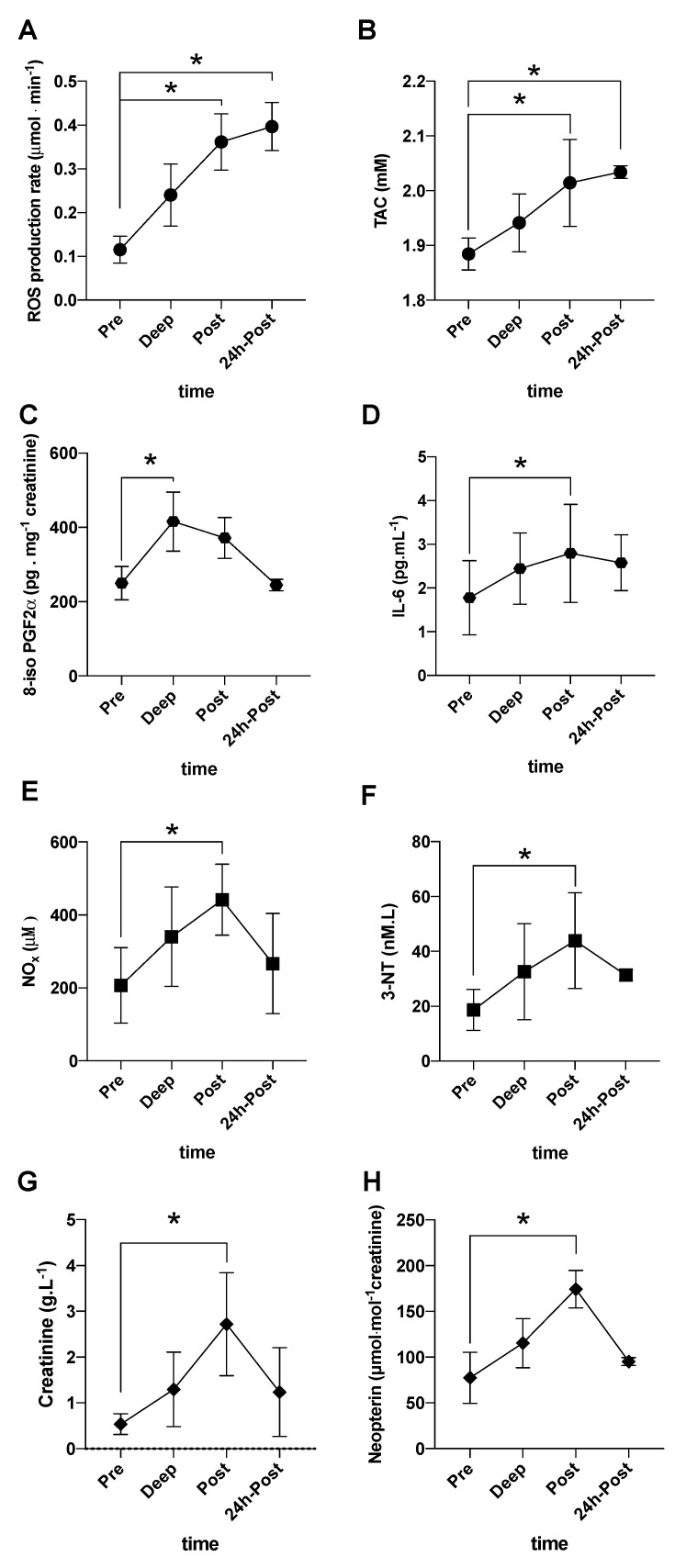
Biomarkers kinetic during saturation diving. Time course of: (**A**) ROS production rate (μmol·min^−1^) and (**B**) antioxidant capacity (TAC−mM) in saliva calculated by electron paramagnetic resonance (EPR); (**C**) 8-isoprostane (8-iso-PGF2α-pg·mg^−1^ creatinine); (**D**) Interleukin-6 (IL-6, pg·mL^−1^); (**E**) nitric oxide metabolites (NOx, µM), and (**F**) 3-Nitrotyrosine (3-NT, nM·L^−1^); (**G**) creatinine (g·L^−1^); (**H**) neopterin (μmol.mol^−1^ creatinine), concentrations detected in urine. All samples were collected at pre-, at deep, post-, and 24 h post-dive in saturation divers. * *p* < 0.05.

Significant increases at post saturation dive of ROS production rate (0.12 ± 0.03 vs. 0.36 ± 0.06 µmol·min^−1^) (Figure 2A), antioxidant capacity (TAC: 1.88 ± 0.03 vs. 2.01 ± 0.08 mM) (Figure 2B), 8–isoprostane (207.0 ± 103.3 vs. 441.8 ± 97.3 pg·mg^−1^ creatinine) (Figure 2C), NOx (207.0 ± 103.3 vs. 441.8 ± 97.3 µM) (Figure 2E), and 3-NT (18.64 ± 7.45 vs. 53.32 ± 18.03 nM·L^−1^) (Figure 2F) were observed.

Moreover, a significant increase at deep respect to pre was found in 8-isoprostane levels (249.7 ± 45.1 vs. 415.7 ± 79.5 pg·mg^−1^ creatinine) (Figure 2C). Finally, significant increases in ROS production rate (0.12 ± 0.03 vs. 0.24 ± 0.07 μmol·min^−1^), and TAC (1.88 ± 0.03 vs. 1.94 ± 0.06 mM) at 24 h post saturation dive were measured (Figure 2A,B) too.

The time of course in Figure 2G,H shows a significant increase of creatinine (0.54 ± 0.22 vs. 2.72 ± 1.12 g·L^−1^) and neopterin/creatinine (73.3 ± 27.9 vs. 174.3 ± 20.53 μmol·mol^−1^ creatinine) levels, respectively, at post-saturation dive. Finally, a significant increase in IL-6 was found at post (1.77 ± 0.84 vs. 2.79 ± 1.12 pg·mL^−1^) (Figure 2D).

## 4. Discussion

In commercial and military saturation diving, workers can live in an underwater habitat or a surface complex. In the latter, divers rest in the living chamber and are transferred to a submersible decompression chamber (the “diving bell”) when they need to reach working depth [28]. Spending time at pressure, divers can extend the available working time without increasing exposure to decompression sickness risk. Still, factors predisposing to this ominous complication have not been ascertained in saturation diving. This is the first study to demonstrate increased ROS production, lipid peroxidation, higher NOx and 3–NT levels, and potential renal damage in such a peculiar environment.

The redox status of human cells is finely regulated, with antioxidant systems always balancing ROS generated by normal metabolism. ROS are usually associated with damages and negative consequences exerted on cells but are also involved in several protective mechanisms, such as immune defense, antibacterial action, vascular tone regulation, and signal transduction [29]. Moreover, a recent research study [17] demonstrated that differential gene expression indicated reduced blood O_2_ transport and increased endogenous antioxidant activity after saturation diving. During prolonged exposure to a confined, hyperbaric, hyperoxic environment, antioxidant supplementation is recommended for saturation divers to reduce oxidized molecules [30]. However, ROS production may overwhelm antioxidant capacity. Antioxidants cannot attenuate nitric oxide signaling—either directly (reaction with nitric oxide) or indirectly (reaction with derivatives, e.g., peroxynitrite)—or react with hydrogen peroxide [31].

The membrane lipid peroxidation process is one of the earliest events in oxidative cellular damage and is associated with fine structure disturbance and subsequent function loss. In the present study, lipid peroxidation markers were increased at deep and post-saturation diving, probably resulting from compression rather than hyperoxia, as previously reported [32]. These results are concordant with those of Suzuki [33], demonstrating a significant increase immediately after a 4.5 MPa saturation dive.

As widely reported, modifications in PO_2_ (hyperoxia/hypoxia) can increase ROS accumulation in the whole body [7,13,14,34,35,36]. Additionally, NO has fundamental roles, especially in regulating peripheral [37] and pulmonary vascular tone [38]. However, NO can react with ROS to generate peroxynitrite (ONOO), adding another source of damage to cells [39].

Indeed, in the present study, the level of 3-NT, the product of tyrosine nitration mediated by reactive nitrogen species such as peroxynitrite anion increased. Otherwise, in our experimental set-up, the growth of circulating ROS, NOx, and 3-NT levels is probably due instead to the prolonged hyperoxic exposure than to exercise. In fact, in a previous study performed by the authors [14], the increased oxidative stress and NOx were both generated after sled-assisted breath-hold dives and breath-hold dives using fins but were higher in the latter.

The levels of neopterin and creatinine can increase during systemic oxidative stress [13,14,40,41].

In the present study, the increase of these biochemical parameters’ concentration was observed immediately post-saturation diving and was associated with a rise in ROS production. Even though this study did not evaluate the chronic or long-term effects of saturation diving on kidney function, the four divers manifested a temporary “impairment of renal function” as a likely physiological or adaptive response to hyperoxia. Hyperoxic acclimatization in saturation is also in line with reports of transient symptoms of hypoxia; therefore, management of oxidative stress is essential for health preservation. The body’s endogenous redox systems generally provide this in normoxia, but diving challenges the physiological balance by inducing ROS production in excess. As reported by Brubakk et al. [7] and confirmed in our study, during saturation diving, hyperoxia, partial pressure changes, and inert gas exchange during decompression are likely sources of excess ROS.

Additionally, we found an elevated concentration of IL-6 in post-dive. IL-6 is the first cytokine released into the circulation [42], reflecting a pro-/anti-inflammatory response. In agreement with Stenvinkel et al., we reported elevated levels of creatinine, oxidative stress, and inflammation [43], suggesting that the systemic effect in diving may be due to hyperoxia, hyperbaria, and exercise at depth.

A statistically significant difference was found in post diving as previously reported [17].

It is worth noting the reliability of the micro-invasive methods adopted in this study, reflecting the oxidative/inflammation systemic response. Such techniques are feasible in paramount situations such as the “deep environments” and, at the same time, allow easier recruitment of subjects for scientific tests.

These results could help to improve commercial, police or military diving, scientific exploration, or even help to prepare astronauts for future space exploration. Therefore, the results can help to understand the mechanism generating distress symptoms caused by elevating of the oxygen levels or other phenomena.

## 5. Limitations

Diving deeper into the water is only for professional saturation divers, and it is a career which is very hard and dangerous. Therefore, there are only a few professionally habilitated saturation divers in the world. It is clear, therefore, that the study suffers from some limitations. Indubitably, the too small number of investigated divers and the high variability among subjects is a limitation, but also the lack of blood sampling during the study, which was a decision made so as to be as little invasive as possible.

Be that as it may, our strength consists in assessing, for the first time, ROS production and antioxidant capacity by EPR, nitric oxide level, and renal function before, during, and after saturation diving, with non-invasive methods.

The main limitation of our study is the small sample. We were forced to include only four subjects due to the scarcity of saturation divers available to take part in the study, and due to technical and logistics constraints. Therefore, results have to be interpreted carefully, and the conclusions cannot be extrapolated to every saturation diver. This being a pilot study, we plan to set international collaborations in the future to increase the number of participants. Nevertheless, this is the first study to assess ROS production, antioxidant capacity, and nitric oxide levels in saturation divers in a real environment.

## 6. Conclusions

Saturation diving is shown to induce an increase in ROS and NO metabolites. Moreover, in this experiment, transient damage in renal function was noted. Future studies are required to investigate the biochemical processes and the clinical correlations consequent to chronic exposure to high pressure.

## Figures and Tables

**Table 1:** Anthropometric and physiological parameters of all saturation divers. Parameters collected from the divers at pre- and post-saturation diving. BMI: body mass index; HR: heart rate; SBP: systolic blood pressure; DBP: diastolic blood pressure; T: temperature.

Subject	Anthropometric and Physiological Parameters									
	Pre					Post Saturation Diving				
	1	2	3	4	Mean ± SD	1	2	3	4	Mean ± SD
Age	32	48	44	39	40.7 ± 6.9	-	-	-	-	-
Weight (Kg)	71.5	71.7	82.5	87.8	78.4 ± 8.1	69	68.7	79.8	84.7	75.5 ± 7.9
Waist (cm)	76	84	89	93	85.5 ± 7.3	72	83	88	93	84.0 ± 8.9
Hip (cm)	92	92	96	105	96.2 ± 6.1	92	88	94.5	105	94.9 ± 7.3
BMI (kg·m^−2^)	23.9	26.3	26.6	28	26.2 ± 1.7	23	25.3	25.7	27	25.2 ± 1.6
HR (BPM)	80	82	89	104	88.7 ± 10.8	54	60	76	68	64.5 ± 9.6
SBP (mmHg)	130	115	110	135	122.5 ± 11.9	140	110	105	150	126.3 ± 22.1
DBP (mmHg)	76	75	65	75	72.7 ± 5.2	95	75	80	80	82.5 ± 8.6
T (°C)	35.9	35.5	36.3	36.3	36.0 ± 0.3	35.9	34.6	35.9	35.8	36.5 ± 0.6

**Table 2:** Antioxidants supplementation. Daily dosage of supplementation, oral use.

Subject	Supplementations Before Bells	Supplementations/Die During Saturation Diving
**1**	Magnesium 1 tablet/die	Vitamin C 1000 mg
	Creatinine 2 g	Branched-chain aminoacids 8.1 4 tablets
**2**	Branched-chain aminoacids 8.1 4 tablets	Vitamin C 1000 mg
		Vitamin D 3 drops
		Complex Vitamin B 1 tablet
**3**		Vitamin C 1000 mg
		Multicentrum 1 tablet
		Engystol 1 × 3 tablets
		Immunosempre 1 tablet
**4**		Vitamin C 1000 mg
		Branched-chain aminoacids 8.1 6 tablets

## Abbreviations

EPR	electron paramagnetic resonance
3-NT	nitrotyrosine
NO	nitric oxide
NOx (NO_2_ + NO_3_)	nitric oxide metabolites
ROS	reactive oxygen species
TAC	total antioxidant capacity
PO_2_	partial pressure of oxygen
O_2_	oxygen
